# Supplementary material for: The AltR transcription factor responds to plant thiosulfinates to regulate gene expression in a bacterial pathogen of onion
Source: PLoS Pathog. 2026 Apr 30;22(4):e1014198. doi: 10.1371/journal.ppat.1014198 (PMC13178969; doi:10.1371/journal.ppat.1014198)
Supplement: S1 Fig — Quantitative analysis of relative allicin ZOI area for wild-type (WT) and PaltRmod box strains. Data represents three biological replicates, each with three technical replicates, relatively compared with the average area of WT, analyzed by one-way ANOVA on ranks followed by Dunn’s multiple-comparison test (p < 0.01). (DOCX) [file ppat.1014198.s001.docx]

**
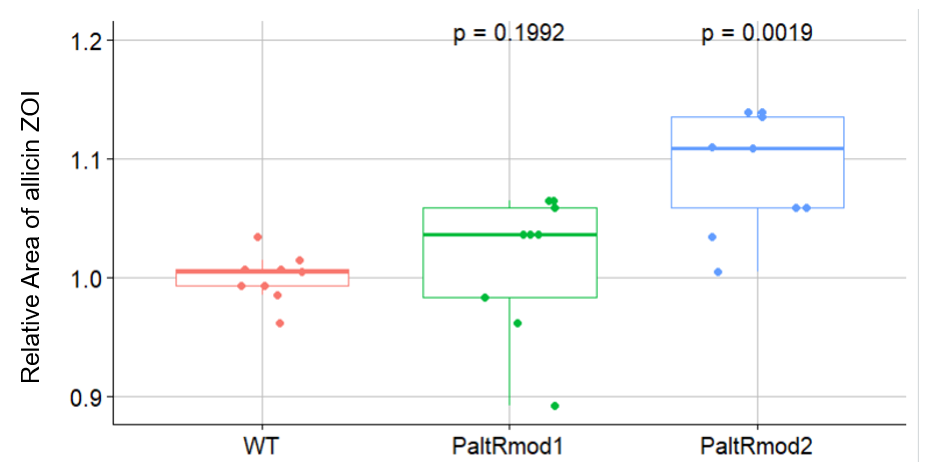
**

**Fig S1. ZOI comparison between the modified altR box strains.**

Quantitative analysis of relative allicin ZOI area for wild-type (WT) and PaltRmod box strains. Data represents three biological replicates, each with three technical replicates, relatively compared with the average area of WT, analyzed by one-way ANOVA on ranks followed by Dunn’s multiple-comparison test (p < 0.01).
