# Supplementary material for: The AltR transcription factor responds to plant thiosulfinates to regulate gene expression in a bacterial pathogen of onion
Source: PLoS Pathog. 2026 Apr 30;22(4):e1014198. doi: 10.1371/journal.ppat.1014198 (PMC13178969; doi:10.1371/journal.ppat.1014198)
Supplement: S2 Fig — Growth curve in LB media of altR Cys to Ser mutant strains used in Fig 5 to Fig 7. Overnight LB cultures were normalized to OD600 of 0.2 before incubating in a 96 well plate and kinetic measurement of OD600 every 30 minutes for 26 h with a SpectraMax iD3 plate reader (Molecular devices, San Jose, CA, USA). (DOCX) [file ppat.1014198.s002.docx]

**Fig S2. Growth curve of the PNA 97-1R Cys to Ser mutants in rich media.**

Growth curve in LB media of altR Cys to Ser mutant strains used in Fig 5 to Fig 7. Overnight LB cultures were normalized to OD600 of 0.2 before incubating in a 96 well plate and kinetic measurement of OD600 every 30 minutes for 26 h with a SpectraMax iD3 plate reader (Molecular devices, San Jose, CA, USA).
