# Supplementary material for: The AltR transcription factor responds to plant thiosulfinates to regulate gene expression in a bacterial pathogen of onion
Source: PLoS Pathog. 2026 Apr 30;22(4):e1014198. doi: 10.1371/journal.ppat.1014198 (PMC13178969; doi:10.1371/journal.ppat.1014198)
Supplement: S3 Fig — 3 DPI onion scales necrotic symptoms for PNA 97–1 mutants. (DOCX) [file ppat.1014198.s003.docx]

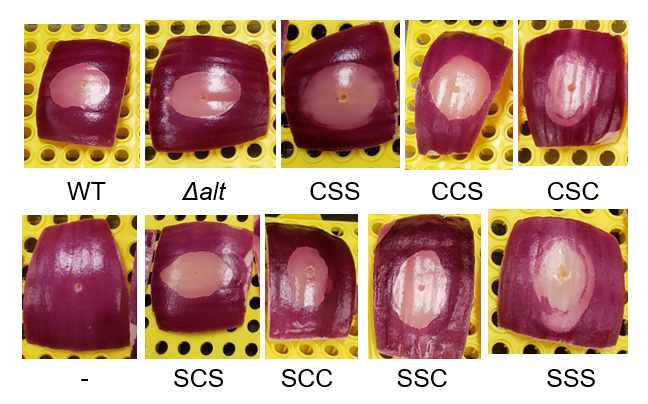


**Fig S3. Necrotic lesions on red onion scales caused by Cys to Ser mutants.** 3DPI onion scales necrotic symptoms for PNA 97-1 mutants.
