# Supplementary material for: The AltR transcription factor responds to plant thiosulfinates to regulate gene expression in a bacterial pathogen of onion
Source: PLoS Pathog. 2026 Apr 30;22(4):e1014198. doi: 10.1371/journal.ppat.1014198 (PMC13178969; doi:10.1371/journal.ppat.1014198)
Supplement: S1 Table — (DOCX) [file ppat.1014198.s004.docx]

**Table S1.** **General cloning, deletion, and labeling strains and vectors used in this study.**

| Species | Strain / Plasmid | Purpose | Source |
| --- | --- | --- | --- |
| E. coli | MaH1 | *attTn7* *pir116* R6K replicon plasmids, DH5α derivative | [1] |
|  | RHO5 | *pir116*, DAP-dependent conjugation strain,  SM10 derivative | [1] |
|  | DH5α | General plasmid cloning strain | [2] |
|  | RHO3 / pTNS3 | DAP dependent conjugation strain SM10 derivative with Tn7 transposase helper plasmid (Amp^R^) | [3] |
|  | DB3.1 / pDONR221 | Gateway BP clonase compatible cloning  vector | Invitrogen |
|  |  |  |  |
| Species | Strain | Plasmid | Source |
| *P. ananatis* | PNA 97-1R (WT) |  | [4] |
|  | PNA 97-1R (Δ*alt*) |  | [5] |
|  | PNA 97-1R (Δ*altR*) |  | [5] |
|  | PNA 97-1R (WT) Tn7PaltRLuxK6 |  | This study |
|  | PNA 97-1R (Δ*alt*) Tn7PaltRLuxK6 |  | This study |
|  | PNA 97-1R (Δ*altR*) Tn7PaltRLuxK6 |  | This study |
|  | PNA 97-1R (Δ*altR*) Tn7PaltRLuxK6 | pBS46::altR | This study |
|  | PNA 97-1R (Δ*altR*) Tn7PaltRLuxK6 | pBS46::altRSSS | This study |
|  | PNA 97-1R (Δ*altR*) Tn7PaltRLuxK6 | pBS46::altRCSS | This study |
|  | PNA 97-1R (Δ*altR*) Tn7PaltRLuxK6 | pBS46::altRSCS | This study |
|  | PNA 97-1R (Δ*altR*) Tn7PaltRLuxK6 | pBS46::altRSSC | This study |
|  | PNA 97-1R (Δ*altR*) Tn7PaltRLuxK6 | pBS46::altRCCS | This study |
|  | PNA 97-1R (Δ*altR*) Tn7PaltRLuxK6 | pBS46::altRCSC | This study |
|  | PNA 97-1R (Δ*altR*) Tn7PaltRLuxK6 | pBS46::altRSCC | This study |
|  | PNA 97-1R (WT) Tn7PaltRLuxK6 | pBS46::altRSCC | This study |
| *E. coli* | MaHI | pTn5/7LuxK6 | [6] |
|  | MaHI | pTn7PaltRLuxK6 | This study |
|  | RHO5 | pTn7PaltRLuxK6 | This study |
|  | DH5α | pDONR221::altR | This study |
|  | DH5α | pUC57::altRSSS | This study |
|  | DH5α | pUC57::altRCSS | This study |
|  | DH5α | pUC57::altRSCS | This study |
|  | DH5α | pDONR221::altRCCS | This study |
|  | DH5α | pUC57::altRSSC | This study |
|  | DH5α | pUC57::altRCSC | This study |
|  | DH5α | pUC57::altRSCC | This study |

**Reference**

1. Kvitko BH, Bruckbauer S, Prucha J, McMillan I, Breland EJ, Lehman S, et al. A simple method for construction of pir+ Enterobacterial hosts for maintenance of R6K replicon plasmids. BMC Res Notes. 2012;5:157. Epub 20120320. doi: 10.1186/1756-0500-5-157. PubMed PMID: 22433797; PubMed Central PMCID: PMCPMC3338088.

2. Liss L. New M13 host: DH5αF’competent cells. Focus. 1987;9:13.

3. López CM, Rholl DA, Trunck LA, Schweizer HP. Versatile dual-technology system for markerless allele replacement in. Appl Environ Microb. 2009;75(20):6496–503. doi: 10.1128/Aem.01669-09. PubMed PMID: WOS:000270552500011.

4. Stice SP, Stumpf SD, Gitaitis RD, Kvitko BH, Dutta B. *Pantoea ananatis* genetic diversity analysis reveals limited genomic diversity as well as accessory genes correlated with onion pathogenicity. Front Microbiol. 2018;9:184. Epub 20180213. doi: 10.3389/fmicb.2018.00184. PubMed PMID: 29491851; PubMed Central PMCID: PMCPMC5817063.

5. Stice SP, Thao KK, Khang CH, Baltrus DA, Dutta B, Kvitko BH. Thiosulfinate tolerance is a virulence strategy of an atypical bacterial pathogen of onion. Curr Biol. 2020;30(16):3130–40 e6. Epub 20200702. doi: 10.1016/j.cub.2020.05.092. PubMed PMID: 32619480.

6. Bruckbauer ST, Kvitko BH, Karkhoff-Schweizer RR, Schweizer HP. Tn5/7-lux: a versatile tool for the identification and capture of promoters in gram-negative bacteria. BMC Microbiol. 2015;15(1):17. Epub 20150204. doi: 10.1186/s12866-015-0354-3. PubMed PMID: 25648327; PubMed Central PMCID: PMCPMC4328036.
