# Supplementary material for: The AltR transcription factor responds to plant thiosulfinates to regulate gene expression in a bacterial pathogen of onion
Source: PLoS Pathog. 2026 Apr 30;22(4):e1014198. doi: 10.1371/journal.ppat.1014198 (PMC13178969; doi:10.1371/journal.ppat.1014198)
Supplement: S2 Table — (DOCX) [file ppat.1014198.s005.docx]

**Table S2. Primers listed in this study for PCR and cloning purposes**

| Name | Sequence (5🡪3) |
| --- | --- |
| attBaltRF | gggg acaagtttgtacaaaaaagcaggct tc ttgaaggattttaaaatgacgattatg |
| attBaltRbspEICfusR | gggg accactttgtacaagaaagctgggt c tccgga ttcctgaggggtagtgtttaaag |
| AltR100_159F | gacctgtcgcacgggc |
| AltR100_159R | tgaaacgagctaccgggcatc |
| altR_outF2 | ccgcattcagattgatggc |
| altR_outR2 | cgtggcagagatgtggtg |
| PNA97-1altRF | atgacgattatgacgcgtgaacg |
| PNA97-1altRR | ttcctgaggggtagtgtttaaagtg |
| Luxpro-UP | attgcactaaatcatcactttc |
| PNA_GlmS_Tn7_down | cgattcacgcgtgagaggc |
