# Supplementary material for: The AltR transcription factor responds to plant thiosulfinates to regulate gene expression in a bacterial pathogen of onion
Source: PLoS Pathog. 2026 Apr 30;22(4):e1014198. doi: 10.1371/journal.ppat.1014198 (PMC13178969; doi:10.1371/journal.ppat.1014198)
Supplement: S3 Table — (DOCX) [file ppat.1014198.s006.docx]

**Table S3.** **Synthesized sequences that were utilized for Gateway cloning and Gibson assembly in this study**

| Name | Sequence (5🡪3) |
| --- | --- |
| PaltR flanks | agatttcacttatctggttggcctgcaaggggaaaaaacgccgattgcttgcc  atagaagggtgtgaaggatacaatctaccaactagtagattgaaggatttt  aaagcggtgacaagtttgtacaaaaaagcaggc |
| PaltRmod1 flanks | agatttcacttatctggttggcctgcaaggggaaaaaacgccgattgcttgcc  atagaagggtgtgaaggatacaatctaccaactaTGCTCGGgaaggatttt  aaagcggtgacaagtttgtacaaaaaagcaggc |
| PaltRmod2 flanks | agatttcacttatctggttggcctgcaaggggaaaaaacgccgattgcttgcc  atagaagggtgtgaaggatacaatAGCAcaactaTGCTCGGgaaggatttt  aaagcggtgacaagtttgtacaaaaaagcaggc |
| attB-altRd100~159-flanks-BbsI | acaagtttgtacaaaaaagcaggctgcgcagggaaacatgatcgacagat  accgaaagggtgagatgctcatggatacagcccgggaaatcgccgcagta  ctgtttcgtgcagatgaagccgctgcgcattgtcgccaccagaacggctgag  gaaaaaacgccgattgcttgccatagaagggtgtgaaggatacaatctacca  actagtagattgaaggattttaaaatgacgattatgacgcgtgaacggctggtt  aatgaagccgagcacctgatgcgggaaaagggctattcagctttcagctatg  ctgacctgtccaagctgattggcatcacgaaggccagtatacatcaccactt  cccgacgaaagaaattctcgggcaggaagtcgtgaagcaggctgtcagtga  cacggttaaccagtttgatcagattgaagcagtacatcaaaaagcggcaga  tcagattacctcttatgtagcgctgtttgaaacgagctaccgggcatcactgtt  gccgctgGGTAGAGTCTTCCCGCTGGAAGACCACACGgaagg  tgccagcgtggtggcccgtgcgacaggtcgggctgaagtgtttaaaataagcc  tgcggcacatccttctcactttaaacactacccctcaggaataatactatgca  tgactggagcaattaccggcaggaattaatgcagcgcctcggcgagctgggc  aagctggcgccggacactatgaaaggcgttgttgcactgggcgctgcaggcaa  taaaaccgacctgctcggtgcaaaggtccgggaactgatagccctggcatgtg  cggtgacaacccgttgcgacggctgcattgcgtttcacgcggaggccgcaatc  aaggccggcgcaacggatgaagaaattgccgaagcgctgggcgttgccatcaa  tctgaatgcgggtgccgctgccgtatattcagcccgaaccctggatgcggtcag  ccagctgcgcggttaaaaaacattaccgcctcctcataaaagggccttgcgg  ccctttttttatttcttcaacccagctttcttgtacaaagtggt |
